# Evaluation of a diabetes decentralization program in rural Madagascar using the RE-AIM framework

**DOI:** 10.1371/journal.pgph.0005936

**Published:** 2026-02-25

**Authors:** Nancy Mugisha, Fanjalalaina Rasoanaivo, Mbolatiana Raza-Fanomezanjanahary, Fiainamirindra Anjaratiana Ralaivavikoa, Tanjaka Andriamanampy, Giovanna Cowley, Haja Ramamonjisoa, Claude Rakotonirina, Tojosoa Rajaonarison, Judith Rahanitriniaina, Mamy Andrianomenjanahary, Lalaina Narovananahary Rakotovoavy, Laura Davis, Bénédicte Razafinjato, Rado J.L. Rakotonanahary, Karen E. Finnegan

**Affiliations:** 1 NGO Pivot, Ranomafana, Madagascar; 2 Department of Global Health, University of Washington, Seattle, Washington, United States of America; 3 Department of Medicine, Harvard Medical School, Boston, Massachusetts, United States of America; 4 Service du District de Santé Publique, Madagascar Ministry of Public Health, Ifanadiana, Madagascar; 5 Association Malgache Contre le Diabète, Antananarivo, Madagascar; 6 Department of Global Health and Social Medicine, Harvard Medical School, Boston, Massachusetts, United States of America; University of Embu, KENYA

## Abstract

Diabetes is a significant cause of disabilities and deaths in Africa. In Madagascar, diabetes causes an estimated 5,000 deaths yearly. There are many barriers to diabetes care in Madagascar, and a key barrier is health systems readiness to provide care, especially in rural areas. To mitigate this, the non-governmental organization (NGO) Pivot and the Ministry of Public Health (MoPH) jointly implemented a diabetes decentralization program in Ifanadiana, a rural district of Madagascar. This program included advocacy; mass screening; training of healthcare workers (HCWs); procurement of equipment, medications and consumables; establishment of a dedicated diabetes care day at primary health centers (PHCs); and support groups for patients. This study evaluated the implementation of this multifaceted diabetes program using the Reach, Effectiveness, Adoption, Implementation, and Maintenance (RE-AIM) framework. Using a mixed methods study design, we evaluated program implementation at PHCs at 3, 6, and 18 months of implementation. Overall, 2.1% of people tested during mass screenings were positive for diabetes and 59.3% of people diagnosed with diabetes during mass screenings entered into care at a PHC. There was a fasting blood glucose check at 93.2% of visits, and an average 1.35 days of medication stock out per month. 48.0% patients had their diabetes controlled, ranging from 33.3% at the PHCs with 3 months of implementation to 57.8% at 18 months of implementation. Qualitative data revealed that HCWs adopted various aspects of the program, with suggestions for improvement on the training received. A dedicated day for diabetes management weekly was widely accepted by patients and HCWs. The most difficult aspect of diabetes management for patients was dietary recommendations. This study demonstrated that NGO support enables accessibility of diabetes services even in the most remote areas, and a multi-layered approach to implementation of diabetes diagnosis and treatment is needed for effective care.

## Introduction

The burden of diabetes has been increasing worldwide, although exact numbers vary based on estimation method [1–4]. A systematic analysis of the global burden of disease estimated that 529 million people of all age groups had diabetes in 2022, whereas the World Health Organization (WHO) reported 830 million with diabetes in 2022 [2]. In 2024, another report by the international diabetes federation (IDF) estimated that 589 million adults between the ages of 20 and 79 years were living with diabetes [3]. These various studies show a three to fourfold increase in the burden of diabetes since 1990 when 200 million people were estimated to have diabetes worldwide [2]. While there are some variations in the estimations of the global prevalence of diabetes, there is a consensus that 80% of people living with diabetes live in low- and middle-income countries (LMICs) [1,3].

The toll of death and disability due to diabetes has followed the same upward trend as the increasing prevalence of diabetes worldwide, reflected in the rising disability adjusted life years (DALYs) and age standardized death rates (ASDRs).The age standardized DALYs due to diabetes reached 915 per 100, 000 in 2021, a 38.6% increase since 1990 [1]. Similarly, the ASDR attributed to diabetes increased by 10.8% from 1990 to 2019 [5], reaching 18.5 per 100,000 person-years in 2019. Notably, the ASDR in LMICs is more than double that of high income countries (HIC) [5]. Both DALYs and ASDRs due to diabetes inversely correlate with national income due to a range of factors including minimal healthcare seeking, lack of resources or limited allocation of resources to diabetes services, and unavailable or unaffordable medications [4–6].

Given the increasing burden of diabetes in LMICs, the WHO included technical recommendations on the integration of diabetes care in the primary care setting in LMICs in their publication of a Package for Essential non-communicable diseases (PEN) in low resource settings [7]. Many LMICs implemented the PEN programs to varying degrees [8–10]. In addition to the WHO PEN protocols, various diabetes programs were initiated in individual countries in recent decades, such as South Africa’s integration of HIV and diabetes treatment which reported fewer missed medication refills with integrated care and their technology-enhanced diabetes prevention program with community health workers (CHWs) which demonstrated feasibility of CHW-run prevention programs [11,12], Kenya’s linkage of diabetes treatment with peer microfinance groups which improved diabetes retention in care [13], and Nigeria’s diabetes self-management education program which improved blood sugar control [14].

In Madagascar, diabetes accounted for an estimated 1,051.9 per 100 000 DALYs in 2021, representing a 16.4% increase in diabetes-related DALYs since 1990 [1]. The prevalence of diabetes in Madagascar is estimated at 4%, and caused approximately 4,785 deaths in 2024 [15,16]. The MoPH released an integrated non-communicable diseases (NCD) policy in 2013 and a strategic plan on NCD care in 2017 that both reinforced the urgency to address the growing burden of diabetes [17,18]. However, there are few reported diabetes management programs in Madagascar. Existing epidemiologic studies describe the prevalence of diabetes, such as a screening program in the northern rural district of Ambanja that evaluated the prevalence of auto-immune diabetes [19], or projects describing the complications of diabetes in Madagascar, such as the project by Sitraka et al that evaluated the burden of diabetic foot disease in two urban centers [20].

To our knowledge, there are no previously documented public primary care diabetes programs in Madagascar. This study evaluates the implementation outcomes of a multifaceted diabetes decentralization program in the district of Ifanadiana, implemented by the MoPH in collaboration with the NGO Pivot. The goal of this initiative was to introduce type 2 diabetes services at the primary care level in the district of Ifanadiana.

Here we evaluate the implementation of the Ifanadiana diabetes decentralization program, using the RE-AIM framework [21], to provide evidence-based recommendations on diabetes decentralization in the context of rural Madagascar.

## Materials and methods

### Study setting

Madagascar, located off the coast of southern Africa, is the fourth largest island in the world. 80% of the Malagasy population lives in rural areas, with a majority of the population having to walk more than 10 km to access a PHC [22,23].

**Fig 1:**
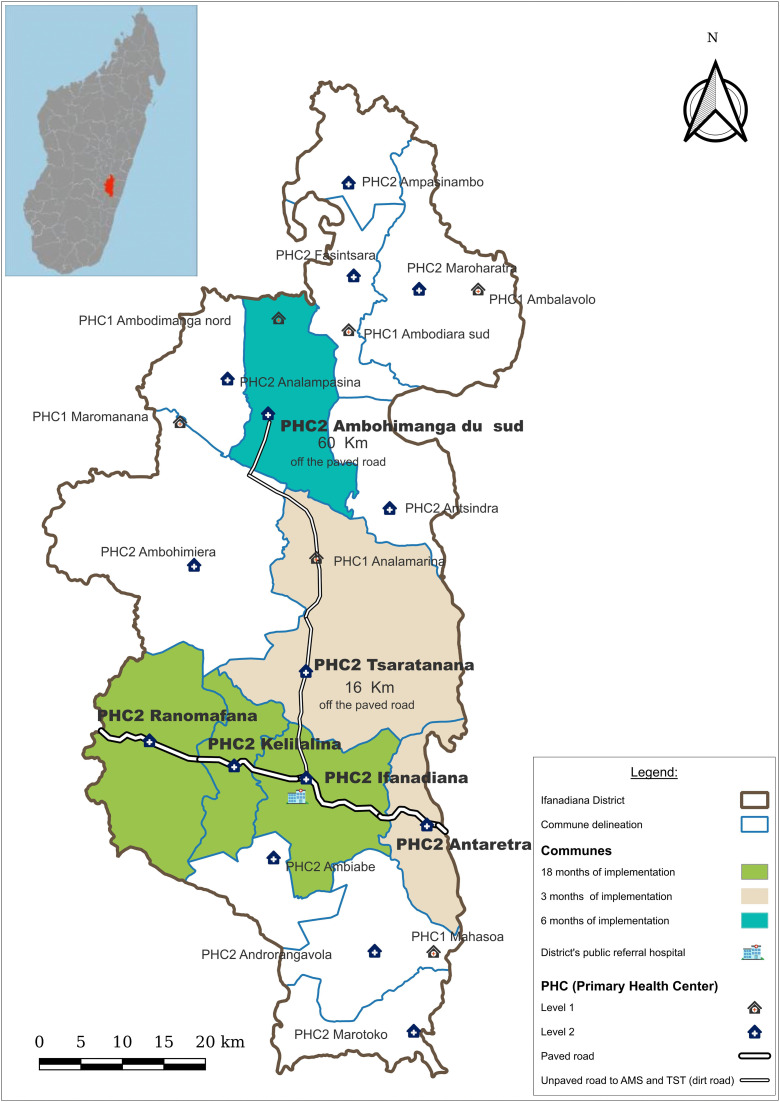
The health system’s network and diabetes decentralization in Ifanadiana district. The upper left panel shows a map of Madagascar, with Ifanadiana district highlighted in red. Map generated with Quantum GIS (QGIS).

The diabetes decentralization program was implemented in the rural district of Ifanadiana, located in Southeastern Madagascar (Fig 1). Since 2014, the NGO Pivot has collaborated with the MoPH to create a model health district by strengthening the public health system [24,25]. The district has one public referral hospital, (*Centre Hospitalier de Référence de District*) which serves as a referral center for its 15 communes [26]. Each commune has a PHC called *centre de santé de base II (PHC II),* with some larger communes having a second smaller PHC called *centre de santé de base I (PHC I)* [26]. Additionally, each commune has CHWs who provide community health education and specific health services.

The population of the district of Ifanadiana faces tremendous geographic barriers to healthcare access. About 75% of the population live more than an hour away from a PHC [27]. Patients travel two to six hours under dry conditions and up to 8 hours during rainy seasons to reach a PHC [27,28]. If a patient requires care at the district hospital, they must first receive a referral from a PHC [26]. Reaching the district hospital located on the paved road can then take multiple days for patients and their families.

Prior to the implementation of the diabetes decentralization program, all patients with diabetes had to reach the district hospital to receive basic diabetes services.

The study sites were six PHCs II which began the implementation of the diabetes decentralization program. Three of the PHCs had implemented the program for 18 months at the beginning of the study. These were the pilot PHCs of Ranomafana, Kelilalina, and Ifanadiana, which were chosen for pilot implementation due to their proximity to the paved road. The PHC of Ambohimanga du Sud was at 6 months of implementation; it was chosen for the next wave of implementation due to the presence of a physician that would mentor the PHC team. The PHCs of Antaretra and Tsaratanana were at 3 months of implementation, and were also chosen for the subsequent wave of implementation due to their accessibility for training during rainy seasons.

### The diabetes decentralization intervention

Immediately prior to each PHC’s program implementation, two or three designated HCWs (nurse, midwife or general practitioner) were trained on the diagnosis and management of diabetes. The training lasted two days and was facilitated by Pivot’s clinical mentor (internal medicine or family medicine physician), Pivot’s NCD manager and the MoPH district’s NCD manager. The training consisted of PowerPoint presentations, chalk talk discussions, and role play sessions. The subjects covered included etiology of diabetes, prevention of diabetes, diagnostic criteria, lifestyle management, and medication management. The trainers also developed a clinical guide for diabetes management, which was distributed during training to be posted at the PHC as an aid to clinical decision-making.

Following training completion, there was a diabetes and hypertension mass screening of the PHC commune during market day, organized by Pivot and MoPH NCD managers. After mass screening, the PHC staff designated one day of the week for NCD care where new and existing diabetes patients could receive routine follow-up appointments. Pivot’s NCD mentor attended NCD day at least once a month for the first three months of implementation at each PHC to mentor HCWs during NCD day’s outpatient visits. On NCD day, patients were expected to not eat breakfast and arrive early in the morning to have their fasting blood glucose (FBG) measured. A HCW who was trained in diabetes management evaluated patients on that day. Diabetes patients were prioritized on NCD day unless there was an emergency. The blood glucose measurement was done during the visit by the HCW. Patients were then prescribed medications and retrieved them from the PHC’s pharmacy the same day.

Pivot donated the first stock of essential medications (metformin and glibenclamide) to launch the program, and provided an ongoing supply of glycemia and urinary testing strips for the program, as these were not on the list of essential medications and consumables of the MoPH and therefore, the PHCs were unable to procure them through the MoPH supply chain. Pivot also simultaneously advocated for an update of the essential list of medications and consumables with the central pharmacy and the Directorate of Pharmacy, Laboratory and Traditional Medicine (DPLTM), a division of the MoPH.

Finally, Pivot’s NCD manager organized monthly support group meetings of patients with diabetes in the three pilot communes only, in collaboration with a Pivot social worker.

### Study design

We evaluated the implementation of the program and patient outcomes with a mixed methods implementation research study. Mixed methods research combines quantitative and qualitative research methods to provide answers that would not be available with either methodology alone [29]. We used a convergent design, with a simultaneous quantitative and qualitative data collection and analysis. We analyzed the quantitative results of the decentralization program alongside the perceptions of stakeholders regarding the program for a comprehensive evaluation of the implementation outcomes. We generated qualitative data through cross-sectional semi-structured interviews of HCWs and focus group discussions (FGDs) with patients. For the quantitative metrics, we used retrospective routine clinical care and logistics data which we extracted from PHC and program records.

Implementation science (IS) is a field that studies factors that influence the effective realization of programs in order to narrow the know-do gap [30]. Health programs are complex interventions that are dependent on various factors for their success, including the context in which they are implemented, the actors that are involved in the implementation, and the technologies necessary for the program. Implementation research and its theories, models and frameworks allow the investigation of several aspects of an intervention to provide recommendations on context-specific evidence-based practices [31–33]. We used the RE-AIM framework to evaluate implementation outcomes pertaining to the diabetes decentralization program as outlined in Table 1 [21].

**Table 1:** Implementation metrics by domain of the RE-AIM framework [21].

RE-AIM domain	Quantitative metric	Qualitative metric
**Reach**	Number of people screened	
	Place of residence of people screened (distance from PHC)	
	Percentage of diabetes positivity during screening	
	Percentage of patients diagnosed who initiated treatment	
	Number of people diagnosed during subsequent PHC routine screening	
	Mean number of visits per patient by phase of implementation	
**Effectiveness**	Percentage of patients with diabetes controlled defined as the average of the previous 3 months FBG ≤ 7.0 mmol/L.	
**Adoption**	Percentage of missed appointments	HCWs’ engagement in the implementation
**Implementation**		
Fidelity	Frequency of FBG measurements	
	Percentage of appropriate medication titrations according to the diabetes management guide	
Acceptability		HCWs’ perceptions about the program’s training
		HCWs’ perceptions about NCD day
		Patients’ perceptions of diabetes management
		Patients’ perceptions of NCD day
Feasibility	Mean stock out days of medications and consumables per month	
	Percentage of missed FBG measurement due to malfunctioning equipment	
**Maintenance**	Percentage of retention in care: seen in clinic in the previous 3 months	
	Average attendance of support groups	

### Data collection

We conducted 13 semi-structured individual interviews with HCWs and five FGDs with patients, with a total of 19 patients participating in the FGDs. The number of participants in the FGDs varied from two to nine. We used purposive sampling for the selection of participants on NCD day. Every trained HCW and every patient who was present at the PHC on NCD day was approached for participation. One PHC was excluded from the qualitative component of the study due to accessibility issues at the time of data collection. The authors who were conducting the interviews could not travel from Ranomafana where they are based to the PHC situated in Ambohimanga du Sud at the time of data collection due to heavy rains.

Data collection and recruitment of participants occurred from May 1^st^ to July 31^st^ 2023 at the six PHC study sites. For the qualitative data, a lead research assistant conducted HCWs’ semi-structured interviews and patients’ FGDs in Malagasy while a secondary research assistant recorded the interviews and took notes. Prior to the start of data collection, a panel of researchers reviewed the interview guides for the face validity of questions. The guides were then translated from English into French and Malagasy by a professional interpreter. The interpretation was reviewed by one of the study team’s research assistants for clarity and correctness.

The semi-structured interviews with HCWs focused on the themes of training, participation in NCD day, and use of the clinical guide for decision making (S1 File). FGDs with patients included themes of diabetes management, motivations for medication adherence, and NCD day (S1 File). Following each data collection activity, the qualitative data collection team held a debriefing meeting; no substantial changes were made to the interview or focus group guides during the data collection process. Interviews were approximately 15–20 minutes in length and FGDs were 30–40 minutes.

We extracted quantitative data from the mass screening database, the diabetes patient registers, and stock management sheets. Mass screening data were entered electronically by NGO clinical staff on the day of mass screening as part of routine clinical care; at screening activities, clinicians recorded participant demographics. We extracted the following information from the mass screening database: age, village of residence, profession, medical history, blood glucose, blood pressure, and anthropometric measurements. The routine clinical information entered at each patient’s visit once enrolled in diabetes care at the PHC was found in the PHC’s paper-based diabetes register. It contained each patient’s FBG, blood pressure, medications with dosing, and the date for the next appointment. Routine logistical PHC data were extracted from stock sheets managed by the person in charge of medication distribution at each PHC. All the aforementioned quantitative data were extracted and entered into spreadsheets by the principal investigator.

### Data analysis

Audio recordings of individual interviews and FGDs were transcribed and then translated from Malagasy to English using a consulting professional interpreter. The translation was verified by a member of the study team who is fluent in Malagasy and speaks English.

We analyzed the interview transcripts using an inductive thematic analysis. We chose this inductive method of analysis to find patterns of thoughts and opinions of HCWs and patients regarding the new diabetes programs. Major themes extracted from thematic analysis were PHC organizational flow, difficulties with lifestyle management for patients, lifestyle counseling for HCWs, and patients’ involvement in training.

We conducted statistical tests to compare the differences in means and proportions of quantitative variables using the statistical software R, version 4.1.1 [34].

### Ethical considerations

This study was determined to be exempt from human subjects review by the Ethics Committee for Biomedical Research at the Malagasy MoPH. It was also submitted and approved by the University of Washington’s Institutional Review Board (STUDY00017007). We also obtained authorization to conduct the project from the district’s chief health inspector.

All HCWs who participated in the semi-structured interviews and all patients who participated in the FGDs had a thorough explanation of the project in Malagasy and signed a written informed consent to participate. The consent forms were available in French and Malagasy.

## Results

### Reach

2,627 people were tested for diabetes during mass screening events. 84% of the participants lived within 5 km of the commune’s PHC, and 9% lived 5–10 km away. 54 (2.1%) were positive for diabetes, defined as having a FBG ≥ 7 mmol/L or a random blood glucose ≥ 11.1mmol/L. Of the 54 patients who were diagnosed with diabetes at the mass screening events, 32 (59.3%) presented to their first appointment at a PHC and were started on medication. On average, the patients who presented to their first appointment lived 0.8 km from their PHC, whereas the average distance from the PHC for the 22 patients who did not present to their first appointment was 0.4 km. Twice as many women than men were screened (Table 2), but men had a higher rate of positivity of 2.5% vs 1.8% (p-value: 0.29).

**Table 2:** Diabetes prevalence during mass screenings.

	Positive	Negative	% Positive
**Female**	32	1725	1.8%
**Male**	22	848	2.5%

19 additional people screened positive for diabetes during routine testing at PHCs. In total, 73 patients were diagnosed through mass screening and PHC services. The mean age of the 73 diabetic patients was 54.2 years old (Table 3). The mean BMI for diabetic patients was 23.5 kg/m2, and no patients met the criteria for obesity (defined as BMI ≥ 30 kg/m2).

**Table 3:** Baseline characteristics of diabetic patients.

Characteristic	Results (N = 73)	Comparison
**Sex**		Similar to cross sectional studies in South Africa and Ethiopia that reported 64% and 55.7% [35,36]
Female	58.9%	
**Age (years)**		
Mean age ± SD	54.2 ± 12.7	Similar to a prospective cohort in Guinea-Bissau with mean diabetic patients age 50.6 [37], and a retrospective cohort in Ghana with mean age 55.9 [38]
Median age	57.0	
**Mean Body Mass Index (BMI) ±SD(kg/m2**)	23.5 ± 4.6	Similar to a study in Ethiopia that reported 22.2 kg/m2 mean BMI [36] but lower than a South African study that reported a mean BMI of 31.73 kg/m2 [39]
**Hypertensive**	47.9%	Higher than the 22.8% general population rate of hypertension reported in a prevalence study done in the district of Ifanadiana [40]

According to the program’s diabetes management guideline, the recommendations were weekly visits for patients with FBG 14–33 mmol/L, bi-monthly for patients with FBG 7–14 mmol/L, and monthly for patients with FBG ≤7 mmol/L. However, the frequency of scheduled visits in the diabetes register was once a month regardless of FBG levels. The mean number of visits per patient in the PHCs that were at 18 months of implementation for patients who were enrolled from the beginning of the program was 9.8, ranging from 1.0 to 19.0 visits per patient. For the PHC at 6 months of implementation, the mean number of visits was 5.0, ranging from 1.0 to 8.0 visits per patient. The mean number of visits per patient in the PHCs that were at 3 months of implementation was 2.3, ranging from 1.0 to 4.0 visits per patient.

### Effectiveness

Across all study sites, 48.0% of patients had controlled diabetes, defined as the average of the previous 3 months FBG ≤ 7.0 mmol/L at the time of data collection. For the three PHCs that were at 18 months of implementation, the proportion of patients with controlled diabetes was 57.8% (95% CI: 44.8-70.7) while the two PHCs that were at 3 months of implementation had 33.3% (95% CI: 7–60) of patients with diabetes controlled. The PHC at 6 months of implementation was excluded for this indicator because their glucometer was non-functional for several months.

### Adoption

Overall, 20.0% of scheduled appointments were missed. At the PHCs that were at 18 months of implementation, 19.2% of scheduled appointments were missed. At the PHC that was at 6 months of implementation, there were 18.9% missed appointments, whereas the PHCs at 3 months of implementation had 33.3% missed appointments. This indicator excluded one PHC due to missing information in the diabetes register.

Adoption of the program by HCWs was strong across all PHCs. In terms of engagement in diabetes management, HCWs reported that there is always a trained provider to consult with diabetes patients. If the designated NCD HCW was absent, they ensured that someone else who is trained received diabetic patients.

HCW-PHC#4

*“... it’s the two of us who attended the training. If I were missing, the midwife replaces me.”*

HCW-PHC#1

*“There is always someone who receives patients because there are five people who participated in the training.”*

The strategy of having a specific NCD day was especially appreciated by HCWs who reported that having a designated day allows better organization for HCWs and patients, as well as an opportunity to do group counseling on diabetes management.

HCW-PHC#3

*“The advantage is well-organized work. We always make them aware that Thursday is the day which is specialized for NCD day so the patients are accustomed to it.”*

### Implementation

#### Acceptability.

Acceptability of the program varied across its different components. Patients’ perspectives on NCD day were favorable, especially because they were given priority attention before other patients since they were expected to fast for their FBG check. They also appreciated that there was a specific day dedicated for the monitoring of their health.

Patient-PHC#5

*“It’s fine for me. If I can’t come in the morning, I come here in the afternoon or I come here early and they receive me first.”*

Patient-PHC#2

*“It’s a day which is specially destined to monitor our health. It’s an advantage for us. They establish Fridays as the day for a check-up, and it’s up to us to follow that, which is why it’s not a problem for me.”*

Patients revealed a good understanding of what it means to live with a chronic disease and the benefits of taking medications, but the thought of having to take medications for a lifetime and dietary recommendations were daunting for some.

Patient-PHC#2

*“...diabetes is like clothes, we have to wear it, and the medications are like soap, to wash the clothes.”*

Patient-PHC#5

*“The sentence I didn’t like at first was being told: “You have to take it until you die,” when I complained about the medication but I should take it until I die so I can’t do anything with that.”*

Regarding training, opinions were divided, with some HCWs relaying that training length was sufficient while others wished it was longer. They also gave specific suggestions to bring diabetic patients to the training sessions for practical experience in engaging with patients, and to elaborate on dietary recommendations for diabetic patients.

HCW-PHC#4

*“For Diabetes it should be for 4 days because, we should practice directly at the PHC but most of us took the diabetes test to understand the process of the patient, but the participants should interview patients who are already affected to understand the reality of the disease.”*

HCW-PHC#2

*“My suggestion about this training is that it should be more detailed because the patients have their own symptoms so we should do a bit more case study. Or we should practice with diabetes patients.”*

HCW-PHC#3

*“Generally, it is sufficient but it’s also insufficient because the training’s period was short. For example, the patient’s dietary regimen wasn’t thoroughly explained, but only in a general manner.”*

In regard to dietary recommendations, patients generally stated that they understand what they should eat, but sometimes have difficulty adhering to it, especially when the recommendations restrictions are the only foods available.

Patient-PHC#5

*“Considering the sensitization of diet, we should not eat carbohydrates but sometimes, we had to eat cassava because of life. There are some changes in our life because we should persevere to pay attention to our diet but sometimes we can’t, we eat what we have.”*

Regarding the program’s clinical guide for diabetes management, HCWs reported that they use it and rely on it to make medical decisions when they are consulting diabetes patients, as they cannot commit everything to memory.

HCW-PHC#1

*“For me, it’s a memory aid and it helps me.”*

HCW-PHC#3

*“It helps a lot because it’s the protocol to follow. The other advantage is that it’s not known by heart, it helps to make a decision or to know all things to do.”*

#### Fidelity and feasibility.

There were 399 total visits at all the PHCs at the time of data collection. A FBG was checked at 372 of those visits (93.2%). Of the 27 visits with missing FBG, 17 (63.0%) instances were due to a non-functional glucometer as noted in the diabetes register.

A review of the diabetes registers showed that the rate of appropriate titrations compared to the program’s clinical guide was 36.8% (95% CI:27.6-46.1) in the PHCs that were at 18 months of implementation to 50.0% (95% CI:21.7-78.3) in the other PHCs, suggesting that the fidelity of clinical guideline implementation was not optimal (Table 4).

**Table 4:** Quality of care and health systems functioning.

	18 months of implementation	6 months of implementation	3 months of implementation
**Mean stock out days per month**			
Metformin	2.70	0	1.35
Glibenclamide	0	0	0
Glycemia test strips	0.2	NR*	NR
Urinary dipsticks	0	NR	NR
**Rate of appropriate titrations (%)**	36.8%	Excluded**	50%
**Visits with missing/malfunctioning glucometer(%)**	1.4%	40.0%	0%

*NR: not recorded.

**Excluded due to high rate of missing FBG.

We also evaluated health systems functioning factors, such as availability of medications and testing strips. Documentation regarding testing strips was not available at three PHCs for blood glucose testing strips and four PHCs for urinary dipsticks, which reveals a gap in the fidelity of documentation practices.

### Maintenance

**Fig 2:**
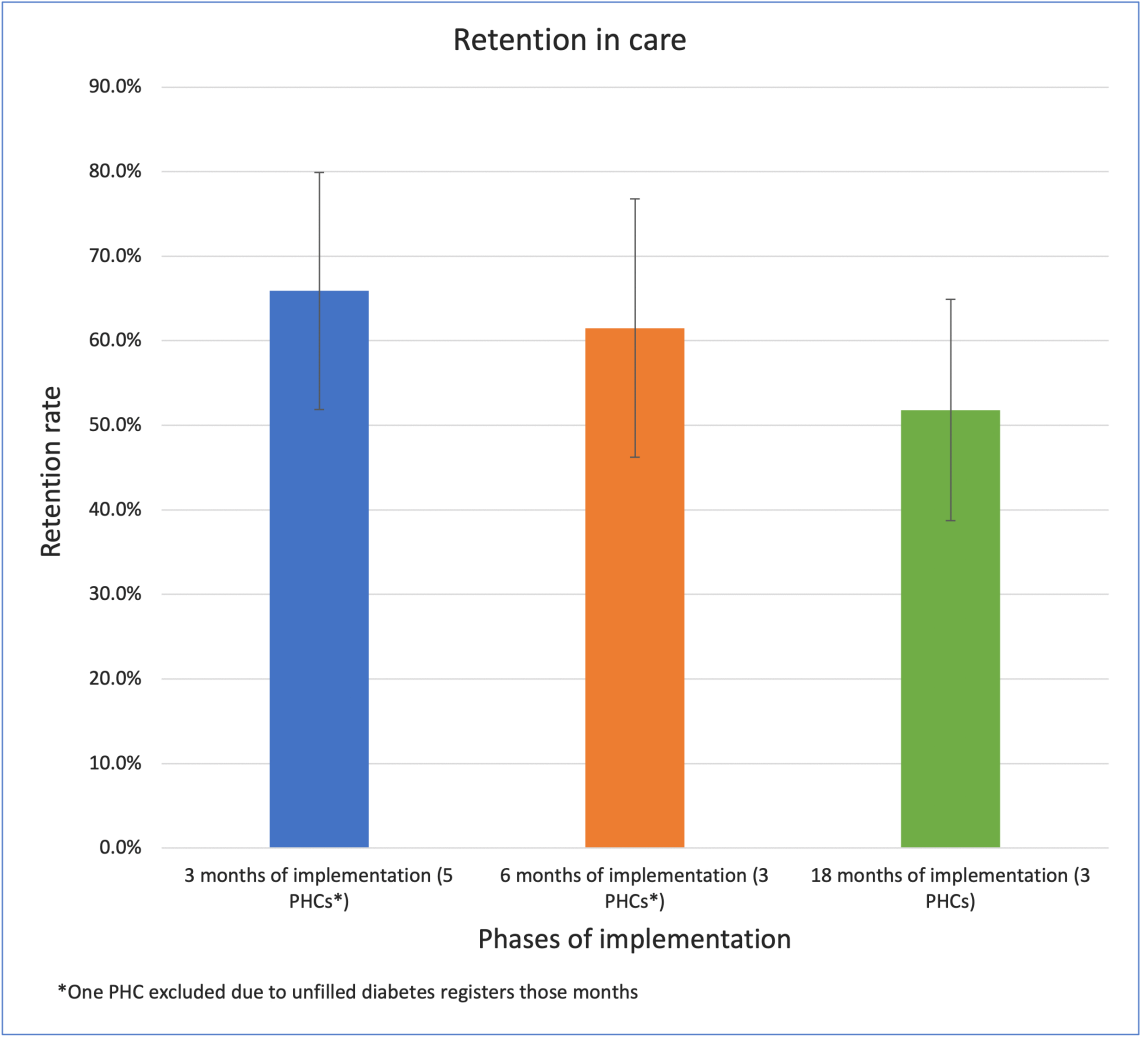
Retention in care. Proportion of patients attending clinic with time of implementation.

We evaluated the sustainability of this program using indicators of retention in care and the average attendance of support groups. Retention in care appears to decline over time (Fig 2), although there is no statistically significant difference (chi-square = 2.1769, degrees of freedom = 2, p-value = 0.34).

**Fig 3:**
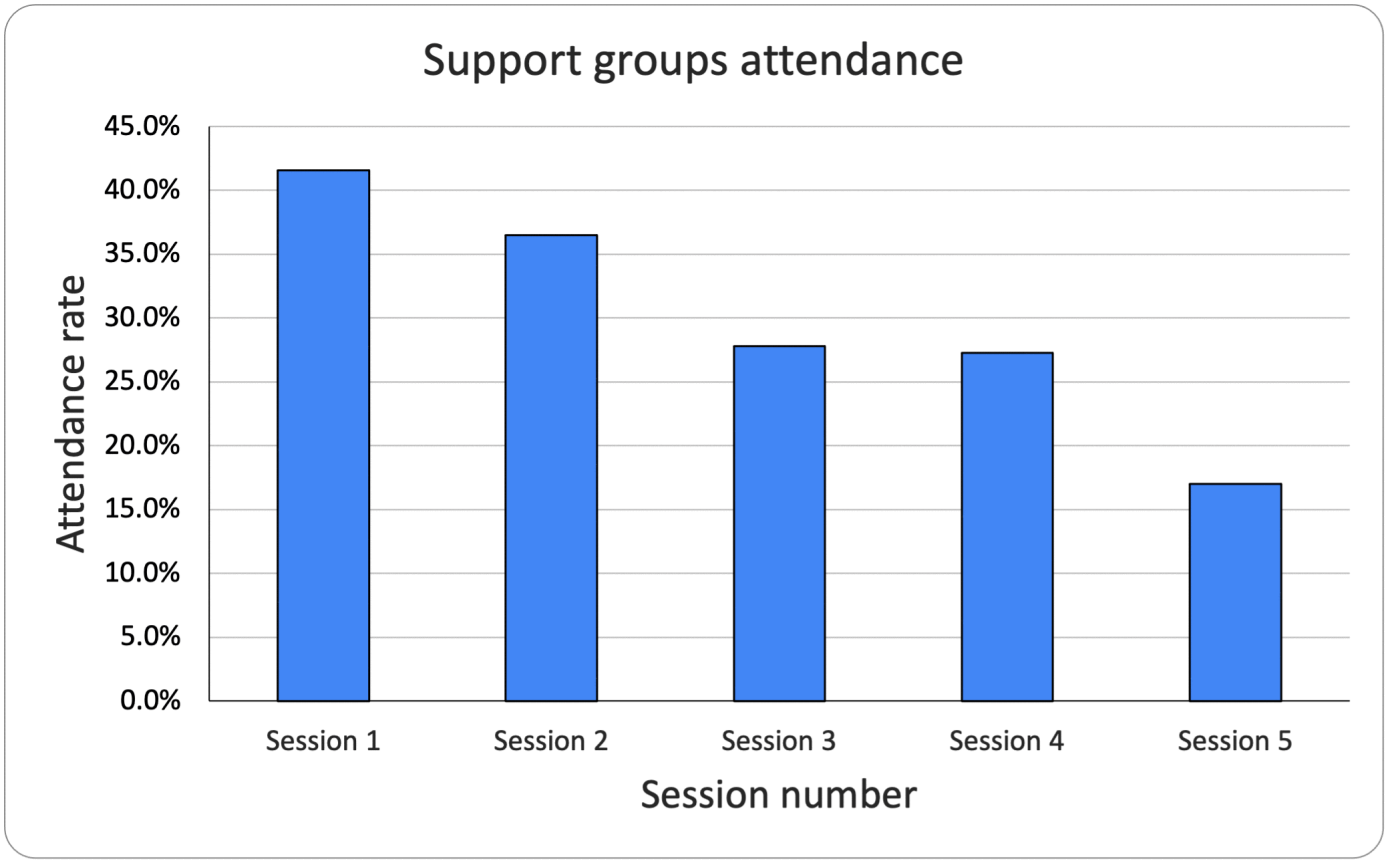
Support groups attendance. Proportion of patients attending support groups at the pilot PHCs over time.

Support groups were only implemented at the three 18-month pilot PHCs for the first five months of program implementation with one group held each month at each PHC. Patient’s attendance at those sessions decreased each month, bringing into question the sustainability of this specific aspect of the program (Fig 3).

## Discussion

This study demonstrated that diabetes care decentralization in rural Madagascar was adopted and acceptable with HCWs and patients. The program reached diabetic patients in remote communes and was feasible for frontline health workers to implement at PHCs after less than one week of training. However, there were gaps in the fidelity to clinical protocols and the sustainability of the program.

The strategy of having a specified NCD clinic on an established day each week was the most unanimously accepted feature of the decentralization program. This is a strategy that has also been successful in other countries such as Rwanda and Malawi, where it allowed optimal organization for the follow-up of NCD patients [41,42]. In our study, HCWs embraced this NCD day and made themselves available on those days, indicating the need to involve non-physicians in the delivery of NCD services in an effort to fill the gaps of physician shortages. This strategy of task-sharing, which is widely practiced for HIV care delivery, has also proven to be efficient in increasing access to NCD care for many sub-Saharan African countries [41,43].

Although the training program was acceptable to HCWs, they also highlighted that the content on lifestyle changes was not sufficient for them to provide comprehensive counseling to patients. This was also reflected in patients’ responses, as they reported having a hard time adhering to dietary recommendations, especially given the scarcity of choices in remote communes. In both high- and low-income countries, dietary adherence is a challenge for patients living with diabetes or other NCDs [44–47]. However, there are some particular challenges for patients in rural areas of LMICs, including space constraints for storage when people live in crowded areas, a lack of reliable electricity which limits cold storage, and the nutrition transition occurring with increasing urbanization [48]. Some studies have reported successful interventions to improve adherence to lifestyle changes for people living with NCDs, such as couples-focused training, using educational videos for dietary recommendations, or teaching diverse culinary techniques [45,46,48,49]. We recommend NCD program managers to consider some of these evidence-based approaches to improve training and counseling on lifestyle changes.

Another aspect of training that was perceived as a weakness was the absence of patients during the training sessions. There were simulated patient encounters in the sessions, however, HCWs suggested that future training sessions should involve real patients. In addition to providing authentic interactions for different scenarios, patients also add valuable input on what needs to be taught as a Ugandan study showed [50].

Diabetes control, which was the effectiveness parameter for the program, is a difficult outcome to achieve in any context, as it requires patient adherence to lifestyle guidelines and medications, as well as providers adjusting medications appropriately. The 48.0% rate of control found in our study is higher than some studies from Mozambique, Uganda, India, and South Africa, which ranged between 19.3% and 42.8% [51–55]. However, an evaluation of a diabetes decentralization program in Bangladesh reported a 69% control rate [56]. There are some key differences between the decentralization program in Bangladesh and our program. The dispensation of medications happened at the community health level in Bangladesh while our program dispensed medications at the PHC. Uncontrolled patients were referred to a sub-district clinic staffed by a physician in the Bangladesh program for medication titration, whereas all patients were primarily managed by non-physicians in our program. Given the geographic barriers of our context, it would be unrealistic to refer every uncontrolled patient to the district hospital, but there could be a communication system that facilitates telephone consultations to physicians at the district hospital. Geographic barriers also impacted the availability of functioning equipment, as the furthest PHC had missing FBG for 40.0% of visits, compared to less than 2.0% for the PHCs closer to the main road.

Regarding fidelity of practicing according to clinical guidelines, the low rate of appropriate titrations reflected clinical inertia, which is defined as a clinician’s decision not to alter medications when a patient’s clinical data shows that their condition is uncontrolled [57]. There was a discordance between the qualitative interview reports on following the guidelines and the clinical decisions made for patients who had suboptimal glycemic readings, as reflected in HCWs reports on use of the guide compared to the fidelity results of 36.8 to 50.0% of appropriate titrations. Clinical inertia is a challenge that has been noted in many NCD programs, whether clinicians are physicians or non-physicians. An implementation study of a hypertension program in rural and urban clinics in Ghana revealed that 80–90% of patients who needed medication titrations were not titrated [57]. Similar clinical inertia has been documented in other studies done in India, Nigeria, and South Africa [58–60]. This is also true for high income countries [61,62]. Some of the reasons that have been proposed for the widespread clinical inertia are doubts about patients’ medication adherence [63–65], fear of side effects, minimizing the effects of chronic diseases on asymptomatic patients, and a lack of appropriate training on diabetes management [62]. Future efforts should include an exploration of what causes clinical inertia in our setting.

Retention in care proved to be the greatest challenge of the program, for clinic visits and the support groups. This is a common challenge for diabetes care delivery. A systematic review on retention in care for type 2 diabetes in sub-Saharan Africa (SSA) reported 33–49% retention in primary care [66]. Some of the factors contributing to this are costs of seeking care, including travel costs, suboptimal counseling, and health seeking behaviors [66,67]. These factors likely contribute to the rate of retention in our program, except the costs of seeking care as patients do not pay user fees for consultations or medications through the NGO’s financial protection program. There needs to be further evaluation of the reasons behind missed appointments in our program. Of note, research has shown that retention in care for various conditions is better at secondary and tertiary level, with a hypothesis that patients who are followed up at those levels are likely to be more symptomatic or have more comorbidities, leading to a higher healthcare seeking rate [66]. Considering this hypothesis to be true, it re-emphasizes the need for better counseling at the primary care level, to stress the importance of continuous treatment even when a patient is asymptomatic.

Besides the implementation outcomes detailed above, there were interesting findings in the patient characteristics, notably their ages and BMI. The median age of diabetic patients was 57 years old, higher than the general population’s median age of 18 years old [68,69]. It was also higher than the median age of the program’s mass screening participants which was 33 years old. We hypothesize that the etiology of diabetic patients in this study has an age-related metabolic factor. The average BMIs of the people who tested positive was 23.5 kg/m2, suggesting that lean diabetes is predominant in the study population. Lean diabetes refers to a diagnosis of type 2 diabetes with normal or underweight BMI (<25 kg/m2). It has been mostly documented in Asian populations, but has also been noted in sub-Saharan African countries [70–72]. Although the pathophysiology of lean diabetes is not well understood, it has been associated with a history of malnutrition in childhood and low socio-economic status [73]. Given that nutritional deficiencies account for 3,748.1 per 100 000 DALYs for children under five years of age in Madagascar [74], this is an area that should be explored further, as well as researching other risk factors for diabetes in rural Madagascar.

One of the limitations of this study is the lack of testing capacity for glycated hemoglobin (HbA1c), which is the gold standard for diagnosis or follow- up of diabetes control, as it is an indicator of 3 months average blood glucose [75]. This likely affected our results on the effectiveness of the program which looked at the rate of diabetes control. When HbA1C is not available, the WHO recommends combining FBG and post-prandial blood glucose. We were not able to perform post-prandial blood glucose as some patients had to travel long distances to get to the PHC. However, FBG is still an acceptable measure for diabetes control even though it is less accurate than the other two measures.

Another limitation of our study was the lack of qualitative data from the most remote study site which is the furthest from the paved road and may have provided different qualitative information. Finally, some data on the feasibility of the program, based on the availability of medications and consumables may be biased by the fact that the NGO was directly supplying them. Therefore, the data may not reflect the feasibility of such a program fully implemented by the public health system without partner support.

The strengths of this study are the pragmatic nature of it, and the fact that the interviews were done in the local language. As a pragmatic study, it reflected the reality of a rural Africa’s NCD program within the context of a public-private partnership. Conducting the interviews in the local language by people who are trained in qualitative data collection also enhanced the reliability of the qualitative findings. This is the first study, to our knowledge, that describes the implementation of a multifaceted diabetes program in rural Madagascar. Since this study was completed, diabetes services have expanded to 13 of the 15 PHCs II of the district.

## Conclusion

This study demonstrated that a diabetes program that includes capacity building, health systems strengthening through the provision of medications and consumables, patient sensitization and a dedicated consultation day is an effective way of decentralizing diabetes care in rural Africa. It also brought up areas that need to be emphasized when implementing similar programs, such as lifestyle modification counseling for patients and continuous guidance to decrease clinical inertia. This model can also be applied to other NCDs, to continue mitigating NCD related complications and deaths in LMICs.

## Supporting information

S1 File Interview guides. Focus group discussions and semi-structured interview templates.
(DOCX)

S2 File Baseline characteristics of mass screening participants. Characteristics of diabetic patients compared to non-diabetic patients.
(DOCX)
