# Supplementary material for: Evaluation of a diabetes decentralization program in rural Madagascar using the RE-AIM framework
Source: PLOS Glob Public Health. 2026 Feb 25;6(2):e0005936. doi: 10.1371/journal.pgph.0005936 (PMC12935218; doi:10.1371/journal.pgph.0005936)
Supplement: S1 File — Focus group discussions and semi-structured interview templates. (DOCX) [file pgph.0005936.s001.docx]

1. **Focus group discussions**

Participants: Patients living with diabetes who receive care at the primary health center

1. Background
   - Probe: Tell me about how you were diagnosed with diabetes?
   - Probe: What made you get tested for diabetes?
2. Perceptions on diabetes management
   - Probe: Please share your experience about diabetes visits at the PHC
   - Probe : What do HCW discuss with you during your visits?
   - Probe: What are your thoughts on having a specific diabetes day at the PHC?
   - Probe: What do you do if you are not able to come on diabetes day?
   - Probe: What challenges have you faced in trying to make it for diabetes day?
   - Probe: What is your understanding of how you will be managing diabetes in one year? 5 years?
3. Influential factors of diabetes services uptake
   - Probe: What are the reasons that motivate you to attend diabetes control day at the primary health center?
   - Probe: What are the reasons that motivate you to come to support groups?

4. Impact of Diabetes on their daily life

- - Probe: How has your day to day life changed after you were diagnosed with diabetes?
  - Probe: Do you have any issues taking medications every day?

**II. Semi-structured individual interview**

Participants: PHC healthcare workers trained in Diabetes management

1. Tell me about your background in terms of diabetes? Had you received training before you had training with PIVOT?
2. I understand there were a few people that went through the training, did you volunteer or were you chosen to participate?
3. How has the training helped you in managing diabetes?
4. Do you have any suggestions on the training content, setting or length?
5. Do you still see diabetic patients regularly? If not, can you elaborate on the reasons? Is there another trained who sees patients?
   1. Follow up probe: who makes decisions on who sees patients? What criteria do people have to meet to be the ones that see diabetes patients on a regular basis?
6. What benefits do you think having this program has provided for patients? for the health center?
7. Can you tell me about a case that was challenging for you? How did you handle that situation?
8. Have you referred any patients with Diabetes to the district hospital for further management? Is there anything you would change about the referral system?
9. How do you make decisions during clinic visits? (For those seeing diabetic patients, Probe: do you use the written guide? If yes, what are some benefits of having this written guide? What are some challenges of using this written guide?
10. What are your views on having a specific NCD day? Depending on the answer , ask: what are the benefits of having a specific NCD day at the health center? and/or : Is there any downside to having a specific NCD day at the PHC?
11. If we were to start this program in another district, what advice would you for the clinical mentor to work well with the providers?
12. What suggestions do you have for the diabetes program in general?
