# Supplementary material for: Evaluation of a diabetes decentralization program in rural Madagascar using the RE-AIM framework
Source: PLOS Glob Public Health. 2026 Feb 25;6(2):e0005936. doi: 10.1371/journal.pgph.0005936 (PMC12935218; doi:10.1371/journal.pgph.0005936)
Supplement: S2 File — Characteristics of diabetic patients compared to non-diabetic patients. (DOCX) [file pgph.0005936.s002.docx]

**Comparison of mean of quantitative variables between diabetes positive and negative persons during mass screening events**

|  | **Diabetes positive**  **(N=54)** | **Diabetes negative**  **(N=2572*)** | **T test** | **p-value** |
| --- | --- | --- | --- | --- |
| **Mean age** | 53.19 | 34.40 | -9.75 | <0.001 |
| **Mean BMI** | 23.43 | 20.64 | -4.05 | <0.001 |

*Participants MAS-2078 and MAS -1796 (see data repository files) excluded from mean age analysis and mean BMI analysis respectively, due to errors in data entry.

**Comparison of proportion of hypertension between diabetes positive and negative persons during mass screening events**

|  |  | **Diabetes positive**  **N (%)** | **Diabetes negative**  **N (%)** | **p-value** |
| --- | --- | --- | --- | --- |
| **Hypertension** | Yes | 26 (48) | 474 (18) | <0.001 |
|  | No | 28 (52) | 2099 (82) |  |
